# Supercritical Fluid Chromatography of Drugs: Parallel Factor Analysis for Column Testing in a Wide Range of Operational Conditions

**DOI:** 10.1155/2017/5340601

**Published:** 2017-06-11

**Authors:** Ramia Z. Al Bakain, Yahya Al-Degs, Bertyl Andri, Didier Thiébaut, Jérôme Vial, Isabelle Rivals

**Affiliations:** ^1^Department of Chemistry, Faculty of Science, The University of Jordan, P.O. Box 11942, Amman, Jordan; ^2^Chemistry Department, The Hashemite University, P.O. Box 150459, Zarqa, Jordan; ^3^Laboratory of Analytical Chemistry, CIRM, University of Liege (ULg), 15 Avenue Hippocrate (B36), 4000 Liege, Belgium; ^4^Laboratoire Sciences Analytiques, Bioanalytiques et Miniaturisation, ESPCI Paris, PSL Research University, 75005 Paris, France; ^5^Équipe de Statistique Appliquée, ESPCI Paris, PSL Research University, UMRS 1158, 75005 Paris, France

## Abstract

Retention mechanisms involved in supercritical fluid chromatography (SFC) are influenced by interdependent parameters (temperature, pressure, chemistry of the mobile phase, and nature of the stationary phase), a complexity which makes the selection of a proper stationary phase for a given separation a challenging step. For the first time in SFC studies, Parallel Factor Analysis (PARAFAC) was employed to evaluate the chromatographic behavior of eight different stationary phases in a wide range of chromatographic conditions (temperature, pressure, and gradient elution composition). Design of Experiment was used to optimize experiments involving 14 pharmaceutical compounds present in biological and/or environmental samples and with dissimilar physicochemical properties. The results showed the superiority of PARAFAC for the analysis of the three-way (column × drug × condition) data array over unfolding the multiway array to matrices and performing several classical principal component analyses. Thanks to the PARAFAC components, similarity in columns' function, chromatographic trend of drugs, and correlation between separation conditions could be simply depicted: columns were grouped according to their H-bonding forces, while gradient composition was dominating for condition classification. Also, the number of drugs could be efficiently reduced for columns classification as some of them exhibited a similar behavior, as shown by hierarchical clustering based on PARAFAC components.

## 1. Introduction

Supercritical fluid chromatography (SFC) becomes an appreciated separation technique in science due to its capacity to provide fast, robust, and efficient analysis [1]. In addition, this technique is considered as green due to its low consumption of organic solvents [2] that are toxic, expensive, and harmful to environment [3]. Therefore, SFC recently showed great success in many fields, such as the separation and detection of PAHs and petroleum related compounds [4–7], oligomers and polymers [8], food residues [9], unpermitted addition and misuse of dyes in different foodstuffs [10], cosmetics and body care products [11], pharmaceutical separation [12], drug development and discovery [3, 13–15], impurity profiling [16–18], and drug testing [1, 19, 20].

During the last decade, many improvements were brought to SFC instrumentation to make this technique compatible with the majority of columns, including columns packed with sub-2 *μ*m particles [21, 22] since a key point for a separation method in SFC is to choose the proper stationary phase [3]. In practice, method development is often based on trial and error, which consumes effort, time, and materials, and limits the age of column due to the numerous runs required. Thus, it would be preferable to understand the behaviors of the SFC stationary phases by testing the available columns under a wide range of operational conditions, that is, combinations of the parameters that influence the chromatographic response: the pressure, the temperature, the type of the organic modifier associated with carbon dioxide as a mobile phase, and the gradient elution of the organic modifier.

Many studies have been carried out to classify and to test different columns generally in chromatography [3–6, 11, 18, 23–28], and specifically in SFC such as those by Lesellier et al., who were pioneers in SFC [3–6, 11, 23–26, 29]. In [4, 5], they compared varied alkyl-bonded Silica stationary phases to investigate the effect of the length of the alkyl chain (from C4 to C18), embedded groups and fluoroalkyl bonding on the separation of PAHs (polyaromatic hydrocarbons), and benzene and naphthalene derivatives at constant temperature, pressure, and mobile phase composition. West et al. also carried out studies on polar stationary phases (Silica gel, cyano and aminopropyl- (NH_2_-) bonded Silica, propanediol bonded Silica, poly(ethylene glycol), and poly(vinyl alcohol)) to evaluate the influence of various polar-bonded groups and polymers and Silica gel on the separation of a wide range of PAHs at constant temperature, pressure, and mobile phase composition of methanol [5, 6]. In another work, they tested the behavior of 26 aromatic stationary phases based on phenyl and pentafluorophenyl ligands using essential oils of grapefruit and lemon (i.e., acidic and neutral compounds) where temperature was set at 25°C and the outlet pressure was maintained at 150 bar for all columns with methanol as organic modifier [23]. In a recent study, West et al. tested different sub-2 *μ*m columns for the separation of ionizable and nonionizable compounds at a constant temperature and pressure with methanol [29]. Grand-Guillaume Perrenoud et al. evaluated the use of columns packed with sub-2 *μ*m particles in SFC and UHPSFC conditions and provided systematic comparisons of the performance for pharmaceutical compounds such as steroids, benzodiazepines and their derivatives, butylparaben, mefenamic acid, diclofenac, acetaminophen, chlorthalidone, indapamide, papaverine, and noscapine at constant temperature and pressure using methanol as organic modifier [30]. In the field of quantification of cosmetics, SFC approaches were developed for the analysis of cream, glyceryl caprylate in eye liner, and caffeine in eye serum at constant conditions of 35°C and 150 bar with methanol as organic modifier [11].

The retention mechanisms involved in SFC are impacted by different parameters: temperature, pressure, chemistry of the mobile phase, and nature of the stationary phase [31]. The addition of an organic modifier (e.g., methanol, acetonitrile, and ethanol) to CO_2_ also has an influence through the modifier adsorption by the column (mainly methanol) and the solubility of solutes in the mobile phase. The use of an organic modifier results in a better distribution of targeted solutes between phases and could lead to improved separation in a reduced analysis time [3]. However, all the previously published studies were carried out by keeping one or two of these parameters constant, and to date no study changing all the parameters together for column testing and classification has ever been carried out.

In the literature, column classification in SFC has been carried out by chemometric analysis based on linear solvation energy relationships (LSER) [3, 5, 11, 25, 29]. Principal component analysis (PCA) and hierarchical clustering (HC) were employed widely in liquid and/or gas chromatography to identify patterns and relationships between the columns since these methods can handle samples of large sizes and complexities. Therefore, these tools have been used for identification, classification, and qualitative control [32, 33], evaluation of the stationary phases [18, 25, 27, 28], and elucidation of retention mechanisms [27, 28, 34]. Moreover, hyphenated chromatographic instruments such as HPLC-DAD or LC-MS can generate tremendous amounts of data in a short time. Thus, the first classical tool that will come to mind for analyzing complex-many-parameter-data is PCA of two-array data. Accordingly, it is not a surprise to see that numerous chromatographers applied PCA along with HC for many purposes including process of column clustering [35]. When handling a three-way data set as in our case, three options are available [36, 37]:

- Subjecting each single matrix to classical PCA: in this case, score and loading plots will end up with little information due to the small size of the analyzed matrices.
- Unfolding the three-way array along each dimension before running classical PCAs. In that case, the chemical or physical interpretation of the loadings may not be guaranteed.
- Stacking the collected matrices together in one cube and applying multiway methods like PARAFAC to end up with interpretable data in the three dimensions. In SFC, it unfortunately appears that modeling three-way array data for pattern recognition is not popular, especially for pharmaceutical separation. Furthermore, the classification of columns using pharmaceutical compounds in SFC seems to be rare, especially when basic solutes are involved due to the appearance of additional retention mechanisms between the drugs and the columns. To our knowledge, the application for PARAFAC has never been reported in column classification in SFC.

Therefore, we propose here a systematic analysis through PARAFAC and HC strategies of a series of SFC columns in a representative set of experimental conditions to unravel the role of three interacting parameters (temperature, pressure, and gradient of organic modifier) with a representative set of drugs (acidic, basic, and neutral). The retention times of the drugs were taken as response for chromatographic testing and classifications of columns and organized in a three-dimensional array (columns × drugs × conditions). The interrelationships between the structurally different stationary phases will be uncovered and two key pieces of information will be obtained: (1) which of the three experimental parameters studied are most likely to impact the classification of the columns and (2) which column-condition couples could exhibit a similar behavior for the considered mixture.

## 2. Experimental

### 2.1. SFC Instrumentation

An Agilent 1100 series system (Agilent Technologies, Waldbronn, Germany) supplied with an auxiliary Aurora A5 fusion SFC module (Aurora SFC systems Inc., Redwood City, CA, USA) was employed in this study. Carbon dioxide, CO_2_, was passed through a G1312A binary pump using high-pressure mixing. The system consisted of a G1314A variable wavelength detector with a 14 *μ*L high-pressure flow cell (10 mm path), and a G1329A autosampler with a 5.0 *μ*L fixed loop. The working temperatures of 35°C and 53°C of the columns were regulated with a Croco-Cil RS 232 column oven, and 16°C was maintained using Huber Minichiller 280 cooling system (USA Inc.).

### 2.2. Chemicals and Reagents

**Scheme 1:**
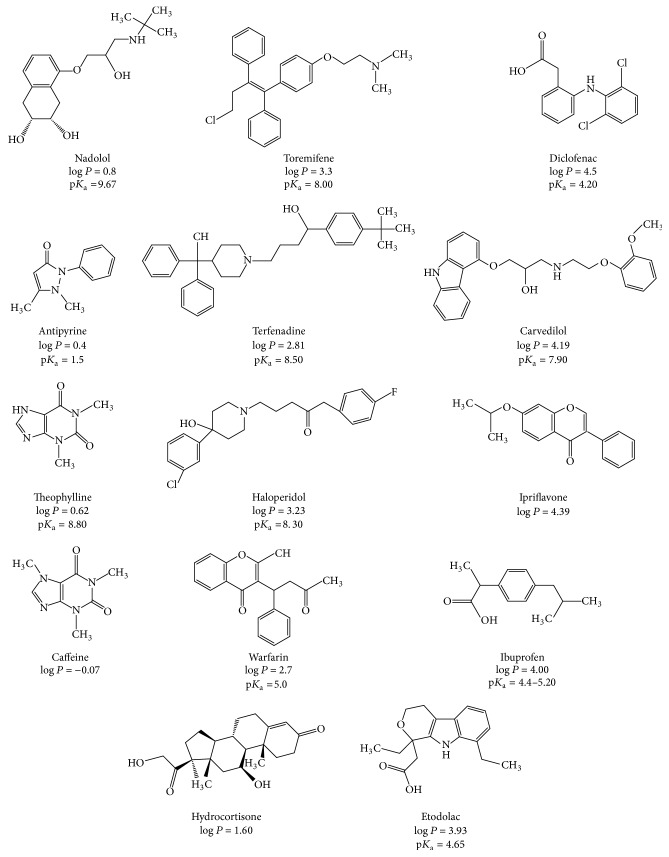
Structures of the 14 drugs with their known physicochemical properties.

A set of 14 drugs including different pharmaceutical families and covering a wide range of p*K*_a_ and log *P* [38] was chosen to test and classify the 8 selected stationary phases.  Scheme 1 provides the full structures of the drugs with their known physicochemical properties.

The 14 drugs were more than 99.5% pure. Carvedilol, diclofenac, etodolac, haloperidol, hydrocortisone, ipriflavone, theophylline, and toremifene citrate were purchased from TCI (Tokyo Chemical Industry Co. Ltd., Tokyo, Japan). Fluka-Sigma Aldrich (St. Louis, MO, USA) provided antipyrine, nadolol, warfarin, and terfenadine. Fagron (Waregem, Belgium) supplied caffeine and ibuprofen. Messer (Puteaux, France) supplied carbon dioxide (purity ≥ 99.995%).

### 2.3. Tested Stationary Phases

**Figure 1:**
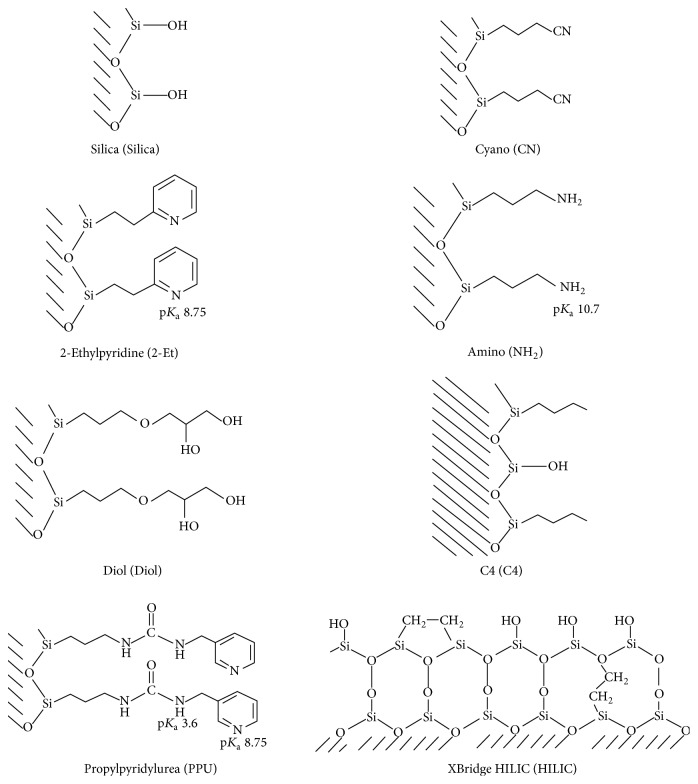
Structures of the eight tested stationary phases.

The testing procedure in this study has been applied to eight commercial stationary phases: XBridge HILIC (HILIC), Silica (Silica), Diol, 2-Ethylpyridine (2-Et), C4, Amino (NH2), Cyano (CN), and Propylpyridylurea (PPU). For comparison purposes, all columns had the same dimensions of 3.0 × 100 mm ID with 3.0 *μ*m particle diameter and were purchased from Princeton SFC (Cranbury, NJ, USA), except for XBridge HILIC, which was purchased from Waters Corporation and filled with 3.5 *μ*m particles. The selected columns were chosen because they are structurally different from each other.  Figure 1 shows the grafted ligands along with the corresponding p*K*_a_ value of the selected columns.

### 2.4. Preparation of Standard Solutions

Stock solutions of the 14 drugs were prepared at a concentration of around 750 ppm in pure methanol. The injection solutions for the chromatographic runs were diluted from the stock solutions in pure methanol in order to provide an UV absorbance around 200–250 mAU (milli-absorbance unit) (the concentrations were within the range ≈100–250 ppm). Between injections, samples were stored at −25°C or less to avoid degradation.

### 2.5. Running Conditions

To analyze samples containing analytes covering a wide range of log *P* and p*K*_a_, gradient elution was preferred to the isocratic elution [3]. The methanol proportion increased from 2.0% v/v (time = 0 min) to 25% v/v (*t* = 5 min) and was then maintained at 25% (*t* = 12 min). After the end of each gradient run, the composition of the mobile phase was gradually set back to the starting conditions for 3 min before running the next injection. The tested drugs have been injected separately at 3.0 *μ*L injection volume twice successively and the average of the retention times was taken as response. UV detection was carried out at 220 nm. All runs were operated at a flow rate of 2.0 mL/min. Brereton's method was applied [39] to set up the Design of Experiment (DoE). We used a 2^3^ full factorial design (chromatographic parameters: temperature, pressure, and % MeOH/min). Four additional central points were provided in the DoE, leading to 12 experiments corresponding to 9 different conditions. The selected levels of each parameter and the codes needed to build the design are presented in Table 1. This design was the same for each of the eight columns.

### 2.6. Software

All chromatographic data acquisition and processing were conducted using Chemstation (rev. B 0402) (Agilent Software, Waldbronn, Germany) software. The statistical analysis was performed with MATLAB (The Mathworks, Natick, MA, USA) 9.0.0.341360 (R2016a) and “the N-way toolbox for MATLAB” [40].

## 3. Statistical Analysis

### 3.1. Data Handling

#### 3.1.1. Structure of the Data

The adopted chromatographic protocol had a good repeatability as evidenced from the repeated measurements of the condition 000 (the central point in the design, see Table 1): Coefficient of variation value on retention times was 1-2% for acidic and neutral drugs and was 7–11% for basic drugs. As indicated in Table 1, the central point was repeated four times. For the subsequent analysis, the mean of the four repetitions was used, reducing the number of experimental conditions to nine. In the following, this three-way array is denoted by *X*, with elements *x*_*ijk*_, for *i* = 1 to *I* = 8 (the number of columns), *j* = 1 to *J* = 14 (the number of drugs), and *k* = 1 to *K* = 9 (the number of experimental conditions). For better legibility in the figures, the column identity is given by the abbreviation of Figure 1, the drug identity by the 3 first letters of its name, and the experimental condition by the abbreviation given in Table 1.

#### 3.1.2. PCA and PARAFAC Analyses

In order to use PCA for the analysis, the *I* × *J* × *K* three-way array must be unfolded in a two-way array for each factor, a *I* × *JK* matrix for the columns, a *J* × *IK* matrix for the drugs, and a *K* × *IJ* matrix for the experimental conditions. PCA consists in rewriting each of these matrices as the product of a matrix of scores and a matrix of loadings and approximating the original matrix by retaining the first components only, typically 2 or 3. The corresponding 2D or 3D score plot enables visualizing the projection of the points (drugs, columns, or conditions), and the composition of the loadings informs about the descriptors that are responsible for the greatest part of the variance. Due to the unfolding of the original three-way array, these loadings combine two factors (column and condition, drug and condition, and drug and column) whose effects get mixed up and are hence difficult to interpret.

A PARAFAC model of the three-way array *x*_*ijk*_ is a decomposition into trilinear components; that is, it is given by three weight matrices *C*, *D*, and *E* [41]. A model with *R* components minimizes the sum of the squared residuals *r*_*ijk*_ in

(1) $$\begin{array}{c} x_{ijk}=\overset{R}{\underset{r=1}{\sum }}c_{ir}d_{jr}e_{kr}+r_{ijk}. \end{array}$$

An advantage of PARAFAC is that, for a given number *R* of components, the minimum is unique, while the optimum number of components can be found by the method of core consistency [37]. In some cases, it can be interesting to interpret one of the matrices as a score matrix and the two others as loading matrices, for example, fluorescence emission spectra measured at several excitation wavelengths for several samples.

Here, each weight matrix plays an equivalent role and will be both considered as weights (on the columns, drugs, or conditions) and used as scores (i.e., in order to visualize the points in a *R* dimensional space or smaller): the chromatographic columns will be plotted using the columns of C, the drugs using those of D, and so forth. Thus, one advantage of PARAFAC over PCA is to introduce only parameters with straightforward interpretation. Another reason to prefer PARAFAC is its parsimony. As a matter of fact, an *R*-component PCA model of an *I* × *J* × *K* array unfolded to an *I* × *JK* matrix possesses *R*(*I* + *JK*) parameters, whereas the *R*-component PARAFAC model has only *R*(*I* + *J* + *K*). Thus, if a PARAFAC model is able to represent a three-way data array, PCA models will also be able to model the unfolded arrays, but since they contain many more parameters, they will be prone to overfitting. To summarize, PARAFAC models are less sensitive to noise than PCA models and provide weights that can be directly related to the different factors of the three-way array.

For PCA, the columns of the three matrices were centered, and we experimented both scaling the columns to unit norm (which amounts to working on the correlation matrix) and not scaling them (the same on the covariance matrix). The results with and without scaling being very similar and since, for PARAFAC, none of the three factors are favored, no centering or scaling was performed, the results shown in the next section are those obtained without scaling. Whereas principal components are orthogonal and ordered by decreasing contribution to the explanation of the variance, PARAFAC weights are not orthogonal, and there is no natural order between the components. We systematically ordered the components provided by the N-Way Toolbox in decreasing order of the squared residuals obtained with each component separately.

#### 3.1.3. Hierarchical Clustering (HC)

Since PCA and PARAFAC are not grouping methods, we classified the drugs, columns, and experimental conditions thanks to HC, using as descriptors,

- for PCA, the scores of the three PCAs performed on one of the three matrices obtained by unfolding the three-way array;
- for PARAFAC, one of the three weight matrices of PARAFAC.

The scores were centered, not normalized, and the Euclidian distance was used.

## 4. Results and Discussion

### 4.1. Classical PCA Analysis by Unfolding the Three-Way Array

**Figure 2:**
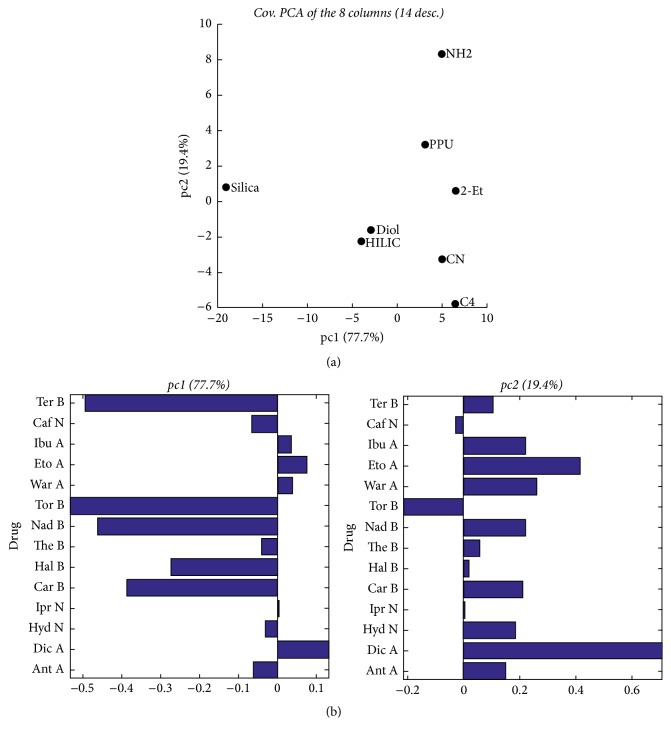
(a) Score plot as generated by PCA for the 8 × 14 matrix (8 columns described by 14 drugs in the central condition only). (b) Loadings of the 8 × 14 matrix (8 columns described by 14 drugs in the central condition only).

Before using PARAFAC, classical PCA was applied to the three matrices obtained by unfolding the three-way array. When studying the behavior of the columns (described by the retention times of the 14 drugs in the 9 experimental conditions), we also worked on the matrix restricted to the central condition (000). Figures 2(a) and 2(b) show the score and the loading plots generated on this matrix by a two-component PCA, which accounts for almost all the variability (97.1%) of the chromatographic data.

The score plot indicates that Diol and HILIC have a very similar behavior, while Silica, which is separated from the others columns by the first component only, displays a very different behavior. As appearing from the loadings in Figure 2(b), (1) basic drugs, terfenadine, toremifene, nadolol, carvedilol, and haloperidol, and (2) acidic drugs, diclofenac and etodolac, have a big weight on PC1 and PC2, respectively, in comparison to the other drugs, which indicate that they play a main role in column classification.

**Figure 3:**
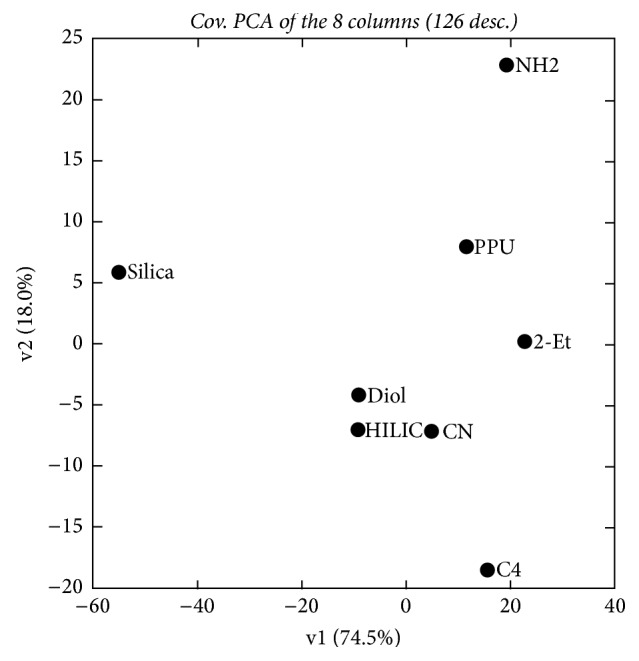
Score plot generated by PCA for the 8 × 126 matrix (8 columns described by 14 drugs in the 9 experimental conditions).

Interestingly, PCA on the whole 8 × 126 matrix describing the 8 columns in the 9 experimental conditions leads to very similar results, as shown by the score plot displayed in Figure 3.

As indicated in Figure 3, the first two principal components also account for almost all the variability (92.5%) of the complete data. Diol and HILIC are gathered close to each other, and CN is not so far from them. Silica remains apart over the nine conditions. Both 2-Et and PPU showed comparable behavior over the nine conditions. NH2 manifested a unique behavior for drugs separation over the nine conditions as it is seen alone in the plot PC2. The same remark is for C4.

**Figure 4:**
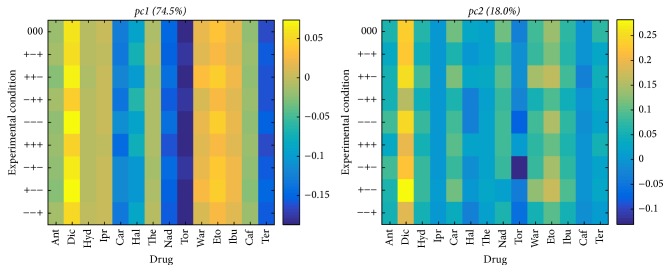
Composition of the loadings of the 8 × 126 matrix (8 columns described by 14 drugs in the 9 conditions).

The composition of the loadings is given in Figure 4: they are reshaped in a 14 × 9 matrix to facilitate the interpretation of the roles of drugs and experimental conditions. Especially for the first component, the similarity of the weights of the drugs across conditions is obvious and is due to the use of a balanced experimental design. A similar behavior is observed when performing the PCA of the drugs: the weights of the columns across conditions also vary very little in the loadings. Thus, it appears that many parameters of the PCAs on the unfolded three-way array are superfluous and that a trilinear PARAFAC model is hence likely to capture the effects of the three factors, columns, drugs, and experimental conditions, while providing more straightforward interpretations of these effects.

### 4.2. PARAFAC Analysis

**Figure 5:**
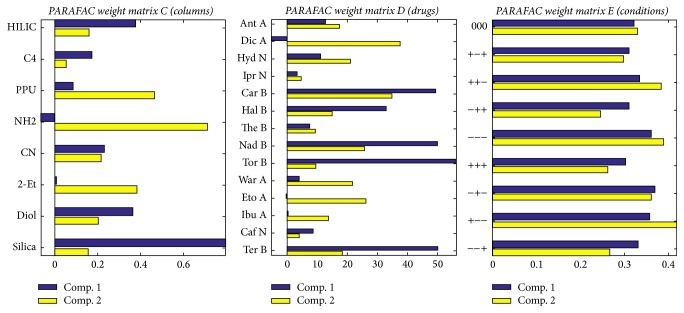
Weight matrices *C* (8, 2), *D* (14, 2), and *E* (9, 2) of the PARAFAC decomposition with two components.

Following the core consistency criterion, a PARAFAC model with *R* = 2 components and explaining 93% of the variance could model the data adequately. The two columns of the three weight matrices *C*, *D*, *E* are displayed as bar plots in Figure 5.

The main conclusions of the two previous PCAs are immediately read on the PARAFAC components: (1) matrix *C*: according to the main component, Silica and, to a lesser extent, NH2 behave very differently from the others. (2) Matrix *D*: according to the main component, the behaviors of the basic and acidic drugs are very different. (3) Matrix *E*: the weights of the experimental conditions vary very little.

The PARAFAC matrices can also be used individually as coordinates similarly to PCA scores for plotting and for clustering using these coordinates as descriptors.

#### 4.2.1. PARAFAC Based Column Classification

**Figure 6:**
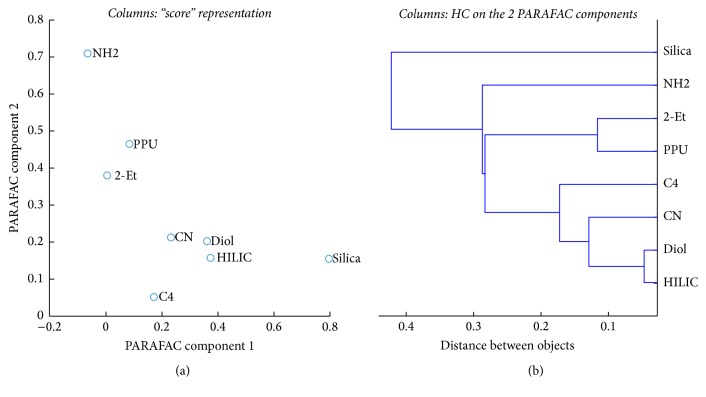
PARAFAC analysis of the chromatographic columns: the two columns of matrix *C* used as *x* and *y* coordinates (a) and HC performed on these coordinates (b).

PARAFAC “score plot” and PARAFAC based HC of the columns are shown in Figure 6.

As two PARAFAC factors were needed to model column mode, then this would support the following issues: (a) there are two main working behaviors for drugs retention by the columns over the nine conditions and (b) the working columns can be divided into two categories according to their attached chemistries. As seen in Figure 6(a), the large variation in the distribution of the 8 tested columns indicated the presence of different retention behaviors. HC was performed on the two first PARAFAC components and provides equivalent information. The retention behavior will be explained as follows.

The nature of the interaction between different stationary phases and probe drugs was explored assuming the following behavior: (a) primary interaction involved between the drug and the main functional group(s) on the surface (Si-OH or grafted ligand for other phases) and (b) secondary interactions between drug and residual -OH groups of the columns. The latter mechanism exists since all tested columns in this study were derived from Silica substrate (see Figure 1). On the other hand, mechanism in SFC depends on the experimental condition and on the physicochemical properties of interacting parts (bulkiness of grafted ligand, chemical states of drugs, and composition of mobile phase) [42, 43]. Therefore, in the current study, the choice behind using MeOH specifically as the organic modifier added to pressurized CO_2_ is helpful for improving the final solubility of solutes, which results in a proper distribution between phases and hence good separation [42]. Clusters of methanol are developed in the mobile phase which improve the polarity of the entire phase [21, 42, 44].

N-grafted bulky ligands of PPU and 2-Et columns provided long retention time due to the H-bonding with basic drugs [45]. This long elution time indicated a favorable interaction between basic drugs and these columns where this behavior is explained as follows: pH of the mobile phase CO_2_/MeOH is known to be 4-5; accordingly, many basic drugs (p*K*_a_ = 7.9–9.67) are positively charged due to protonation of N atom as in our drugs: Nad, Tor, Car, and The as indicated from their structures (see Figure 1). At this pH, the surface of column is mainly in neutral form (or partially protonated for certain phases like PPU) as indicated in Figure 1. Primary interactions as electrostatic interactions are not possible or limited, while the possible interaction would be H-bonding between polar functional groups in basic drugs and the surface -OH groups on the columns [43]. Both 2-Et and PPU phases were clustered close to each other, supposing the close pattern of retention separation mechanism due to the similarity in the bulky nature of attached moieties (see Figure 1).

NH2 was found alone, because the surface is grafted with amine-based ligand and this may suppress secondary interactions between polar drugs and residual polar -OH groups that lead to short elution time for basics solutes in comparison to the other tested columns [44]. Moreover, the NH2 bonded phase should be protonated (NH_3_^+^); the basic compounds are also protonated which explains the repulsion between the basic compounds and the stationary phase, leading to the fast elution of basic drugs.

The fast elution of basic compounds was observed in C4 column due to the unavailable polar functional groups to participate in H-bonding (i.e., the only polar contribution stems from silanol groups) which means weak interaction of drugs with this column [29].

Similar retention behavior due to relative strength of H- bonding was obtained for Diol and HILIC; the two surfaces were characterized by the presence of high concentration of bonded -OH groups which explain the close distance obtained in PARAFAC analysis. Both columns exhibit strong interactions and long retention times, especially HILIC with the silanol groups, which should naturally always contribute to long retention of basic species [29]. The appearance of polar CN with other OH-based-phases was an interesting result as the former column is grafted with a different moiety (see Figure 1). The explanation of this behavior is that PARAFAC depicted the overall behavior of the columns over the nine orthogonal conditions. CN column showed quite long retention times close to those obtained on Diol and HILIC especially for basic drugs; it was hence clustered close to them. This is related to the strong interactions with positively charged drugs. The unpredicted proximity of CN with H-bonding columns was also attributed to the other dominating forces between CN column and the aromatic drugs (*π*-*π* complexation). Protonation of CN ligand in the studied experimental conditions is not possible and this excludes the primary interactions (i.e., electrostatic interactions) in this case. However, strong dipole-dipole interactions may exist, together with interactions with residual silanol groups (favored by the short length of the bonded alkyl chain).

Silica was not close to HILIC and Diol although it provides H-bonding as dominant interaction pathway. This is related to the different behaviors of basic drugs: terfenadine, toremifene, nadolol, haloperidol, and carvedilol on Silica with comparison to HILIC and Diol. These drugs have a big weight on PC2 and are largely responsible for column classification as shown in Figure 2(b), which puts Silica apart from the other columns on the “score” plot. Moreover, the p*K*_a_ of silanols on the regular Silica is around 4, while it is equal to 8 on XBridge HILIC columns, a fact which might explain the difference in behaviors with drugs.

The main conclusion at this stage is that the columns were grouped when they exhibit comparable mechanisms for retaining different drugs including primary and secondary interactions.

Concerning drugs, acidic drugs (Dic, Ibu, Eto, War, and Ant) were eluted faster than basic drugs in all conditions [43]. Neutral compounds showed modest affinity to the studied phases. Neutral and acidic compounds were eluted in a very short time; this can be explained since the interaction of these compounds with the eight tested stationary phases would be attributed to weak Van Der Waals forces and *π*-*π* interaction between these drugs and the grafted ligands attached to the phases. One point that should be mentioned here is that, for the Silica column only, carvedilol and nadolol were not eluted within the frame time of the other columns, leading the Silica column to be clustered away from the others.

Generally, the chromatographic behaviors of drugs would be explained in the light of primary and/or secondary interaction mechanisms. For example, the basic theophylline drug was eluted with nonpolar drugs like (Ipr and Caf) over the tested columns, Antiacidic drug was clustered with neutral Hyd drug which has different chemistry. This unusual behavior can be explained by assuming different types of interactions (i.e., primary and secondary ones). On the other side, secondary interaction was dominant in the retention of the majority of drugs.

#### 4.2.2. PARAFAC Outputs in Condition-Dimension

**Figure 7:**
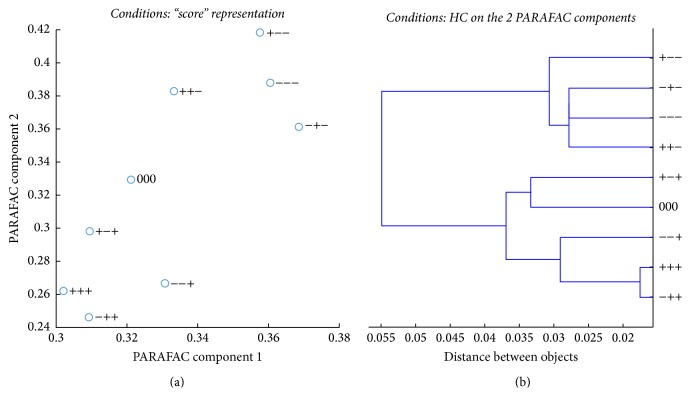
PARAFAC analysis of the experimental conditions: the two columns of matrix *E* used as *x* and *y* coordinates (a) and HC performed on these coordinates (b).

PARAFAC “score plot” and PARAFAC based HC of the experimental conditions are shown in Figure 7.

PARAFAC plots indicated that the tests fall roughly into two distinct categories. Figures 7(a) and 7(b) reveal that the experimental conditions were grouped according to the gradient elution similarity: on one side the conditions of low gradient elution (−) and on the other side the conditions of high gradient elution (+) (including the central point).

The influence of temperature and pressure on clustering of conditions also needs to be commented upon. The close distance between conditions −+− and −−− could be related to the same temperature (16°C) and the same low gradient elution 3.12%MeOH/min but different pressures. Along the same line, conditions +−− and ++− were found in the same cluster as they were operated at the same temperature (53°C) and gradient elution (3.12%MeOH/min). Conditions −−+ and −++ and +++ and +−+ (same gradient and temperature) were found in the same cluster. These results revealed that temperature is more important than pressure. The main conclusion that would be drawn from the earlier discussion is that the studied parameters are important but with different impact; gradient elution of MeOH would have a high influence on the whole process of classifications and on the possible variations of the chromatographic performances of the selected columns followed by the temperature and finally the pressure.

Therefore, according to the results obtained by PARAFAC analysis, the nine experimental conditions could be reduced due to the similarities between some conditions. This will result in lower consumption of solvents, saving of energy, and increasing the lifetime of columns.

#### 4.2.3. PARAFAC Outputs in Drug-Dimension

**Figure 8:**
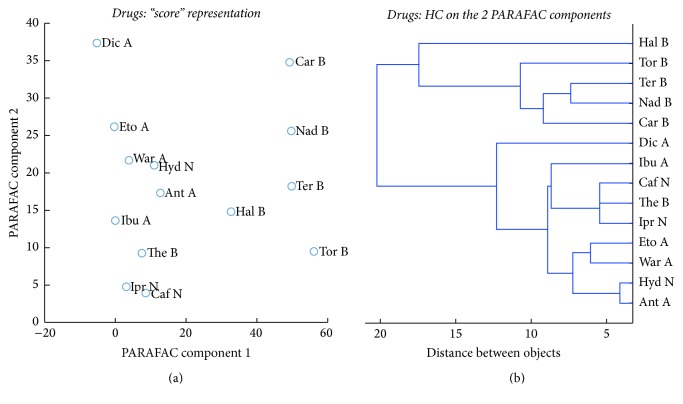
PARAFAC analysis of the drugs: the two columns of matrix *D* used as *x* and *y* coordinates (a) and HC performed on these coordinates (b).

In this part, studying PARAFAC outputs in drug-dimension was addressed to get a clear insight into the entire SFC process. The results are graphically depicted in Figure 8.

Based on Figure 8, 70% of the drugs were grouped according to their acid-neutral versus basic characteristics as follows: group A which is a mix of neutral and acidic compounds (ibuprofen, diclofenac, etodolac, warfarin, theophylline, hydrocortisone, ipriflavone, caffeine, and antipyrine) and group B (basic compounds) (nadolol, terfenadine, haloperidol, toremifene, and carvedilol). Herein, antipyrine is a weak base, which behaved like neutral in our conditions, so it is clustered with neutral hydrocortisone. Theophylline also is a weak acid and behaved as neutral in the current running conditions; thus it was clustered with neutral caffeine and ipriflavone.

**Figure 9:**
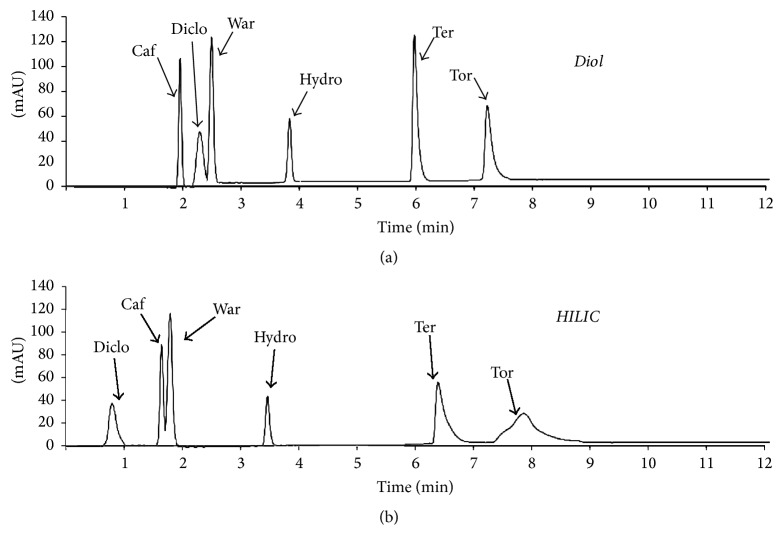
Recorded chromatograms based on PARAFAC outputs at 16°C, 129 bar, and 6.83%/min MeOH gradient elution.

Therefore, based on the above discussion, a subgroup of the 14 drugs might be selected for running future SFC characterization. A chromatogram for drugs carried out in condition −−+ (low temperature and pressure but high gradient elution) is displayed in Figure 9.

## 5. Conclusions

PARAFAC was used for efficient and quick column classification regarding the separation of dissimilar drugs at different operating conditions. To run PARAFAC, a three-way array data (columns × drugs × conditions) was shaped from raw data and modeled to get informative clustering. The wide ranges of the studied chromatographic conditions and drugs were clustered to uncover the complex retention mechanisms often involved in SFC. SFC columns were grouped when they exhibited comparable mechanisms for retaining different drugs including primary and secondary interactions. Regarding the effect of the parameters on column classifications, the gradient elution of % MeOH was the dominating parameter over the temperature and the pressure. Moreover, PARAFAC outputs confirmed that (1) different conditions in SFC could be minimized into only two clusters since some conditions were clustered together (i.e., having the same retention distribution of the dissimilar drugs over the tested columns) and (2) for similar kinds of operational conditions, columns, and experiments, a reduced set of drugs of different chemistries could be involved in the future. Thus, less efforts, time, and materials will be consumed in addition to decreasing column ageing. Finally, the use of PARAFAC was a useful and quick guide for column classification based on the retention time of compounds and for the design of convenient chromatographic future development and optimization method.

## Figures and Tables

**Table 1:** Design of Experiment (DoE) based on Brereton's method.

*T*	*P*	*G*	Codes
(°C)	(bar)	(MeOH%/min)	
35 (0)	175 (0)	5.00 (0)	000
53 (+1)	129 (−1)	3.12 (−1)	+−−
53 (+1)	220 (+1)	6.83 (+1)	+++
16 (−1)	220 (+1)	3.12 (−1)	−+−
16 (−1)	129 (−1)	6.83 (+1)	−−+
35 (0)	175 (0)	5.00 (0)	000
35 (0)	175 (0)	5.00 (0)	000
53 (+1)	220 (+1)	3.12 (−1)	++−
16 (−1)	220 (+1)	6.83 (+1)	−++
53 (+1)	129 (−1)	6.83 (+1)	+−+
16 (−1)	129 (−1)	3.12 (−1)	−−−
35 (0)	175 (0)	5.00 (0)	000
